# A Patient with an Inert Intraocular Foreign Body

**DOI:** 10.7759/cureus.5737

**Published:** 2019-09-24

**Authors:** Tuck Chun Ng, Pei Li Goh

**Affiliations:** 1 Ophthalmology, Miri General Hospital, Miri, MYS; 2 Emergency and Trauma Department, Miri General Hospital, Miri, MYS

**Keywords:** iofb, intraocular foreign body, missed intraocular foreign body, intravitreal foreign body, inert intraocular foreign body, inert intravitreal foreign body, inert iofb

## Abstract

A 29-year-old man was brought to the hospital for treatment after an alleged workplace accident. Initial assessment revealed only mild chest injury and mild confusion, with no other injury. His vision was unaffected with no relative afferent pupillary defect. A computed tomography scan of the brain, performed to rule out brain injury, revealed an incidental finding of a foreign body in the left intravitreal cavity with no other significant findings. Further examination of his medical history revealed that he had experienced a trauma one year earlier, in which his left eye was pierced by a projectile. Immediately post-trauma, his vision had been reduced significantly, but improved over the next few weeks without medical treatment. The current examination of his left eye revealed a small hyperpigmented area on the sclera, representing the point of entry of his previous wound. An encapsulated foreign body was observed in the inferior intravitreal cavity, surrounded by retinal atrophy, and a normal posterior pole. He was managed conservatively without complications. The decision to remove a missed retained intraocular foreign body is complex and depends on multiple factors, including surgical difficulty and the composition, size, and location of the retained foreign body. Removal should be weighed against the possible serious complications of intraocular surgery. If removal is surgically difficult, or the retained material is inert, patients can be managed conservatively with regular monitoring.

## Introduction

An intraocular foreign body (IOFB) is any material that penetrates ocular tissue and is retained within the eye. Due to possible complications, an IOFB is considered an ophthalmic emergency. If the IOFB is toxic, it should be removed as soon as possible. However, if the IOFB is inert, it may be managed conservatively with regular monitoring [1].

## Case presentation

**Figure 1:**
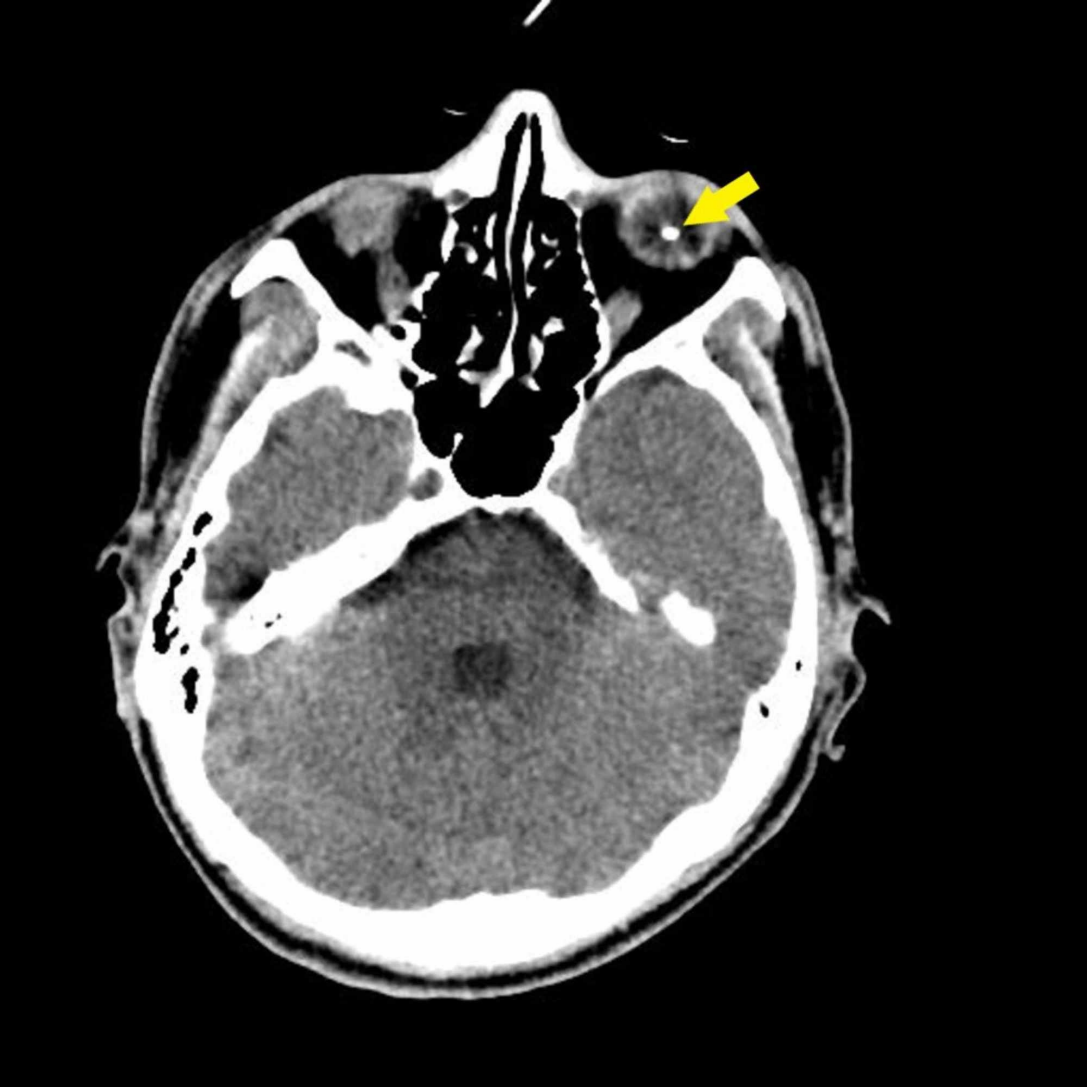
Axial view of the plain CT scan of the brain in this patient, showing a 0.5 cm diameter hyperdense object (arrow), with streak artifacts in the inferior left globe.

**Figure 2:**
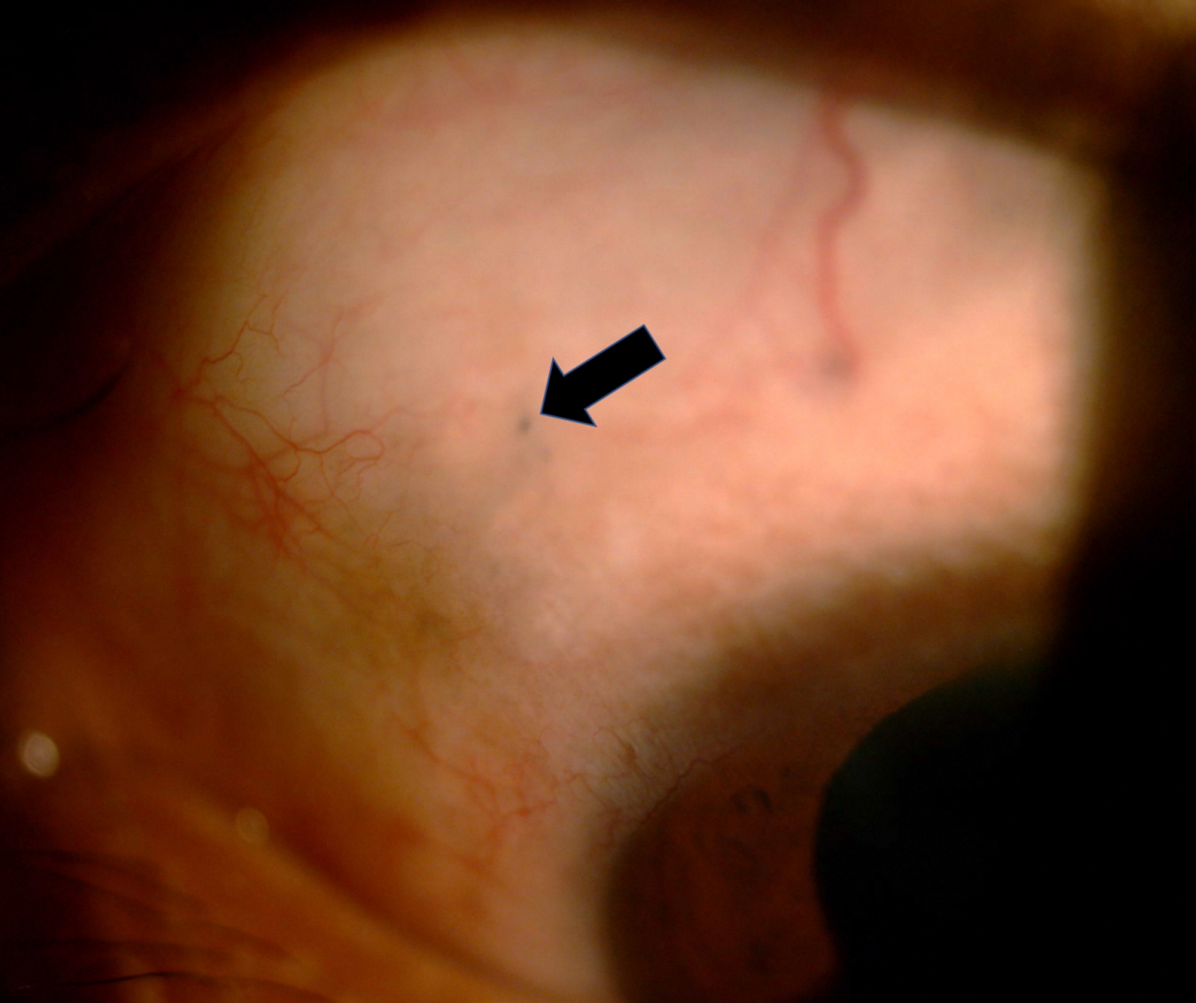
Photograph of the anterior segment of the left eye, showing a small hyperpigmented area (arrow) at the 10 o’clock position of the sclera.

**Figure 3:**
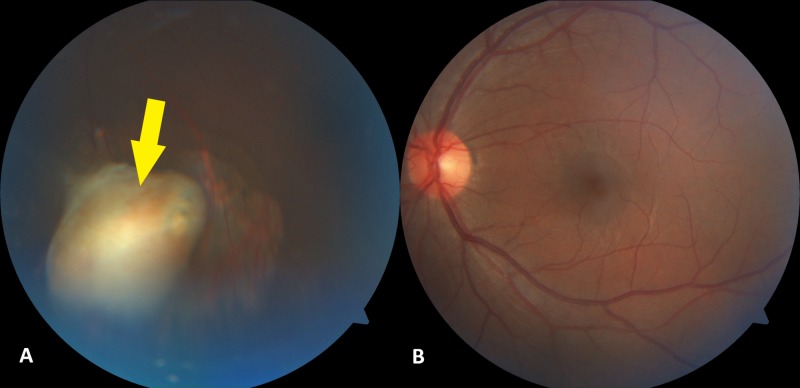
Photographs of the posterior segment of the left eye, showing (A) an encapsulated lesion (arrow) surrounded by areas of retinal atrophy in the inferior intravitreal cavity and (B) a normal-appearing posterior pole.

A 29-year-old man with no known medical illness experienced an alleged workplace accident, during which the back of his trailer landed on his back when he was in a prone position. There was no direct injury to his eyes. Upon arrival at the hospital, he was found to be confused, with a reduced Glasgow coma scale score. There was no other injury other than some bruises on his chest wall. A computed tomography scan of the brain, performed to rule out brain injury, revealed a foreign body in his left intraocular space (Figure 1). His brain was otherwise normal. Further examination of his medical history showed that he had experienced a trauma one year earlier. While he was cutting grass with a brush cutter, a projectile went into his left eye. Immediately after trauma, there was bleeding from his left eye, and his vision was reduced significantly. He did not seek any medical treatment at that time but self-medicated with topical antibiotics. Over the next few weeks, his vision progressively improved, to the level observed before the trauma. Current examination showed that the vision in each eye was 20/30 on the Snellen eye chart, with no relative afferent pupillary defect. His conjunctiva was white with a small hyperpigmented area on the superior nasal sclera (Figure 2), likely representing the point of entry of his previous wound, but the remainder of the anterior segment was normal. Examination of the posterior segment of his left eye revealed a normal posterior pole, along with a yellowish lesion in the inferior intravitreal cavity surrounded by retinal atrophy (Figure 3). The patient was treated conservatively as there was no active inflammation, and the rest of the retina was normal. On subsequent visits, his vision remained good, and there was no sign of inflammation. 

## Discussion

Patients who experience open globe injury with a retained IOFB shortly after trauma have been advised to undergo IOFB removal to avoid retinal toxicity. Some patients with undetected retained IOFB may present with visual loss and chronic ocular inflammation, or they may be detected incidentally, as observed in the present patient [2]. IOFBs can be broadly classified as composed of organic and inorganic materials, with the latter including metals, glass, and plastics. They are also classified as inert or toxic. Following direct mechanical damage, caused by the passage of the foreign body through the ocular tissue, any subsequent complications are influenced by the composition of the IOFB. Compared with inert IOFBs such as glass, higher rates of endophthalmitis have been observed in patients with organic IOFBs, and higher rates of metallosis in patients with metallic IOFBs [3]. Other complications include secondary glaucoma, retinal detachment, proliferative vitreoretinopathy, and sympathetic ophthalmia [4]. The present patient was treated conservatively, both because he had no visual symptoms and because he did not desire surgical intervention.

Depending on their composition, shape, and location, IOFBs can be asymptomatic for many decades [5]. The IOFB in our patient was asymptomatic for over one year. Therefore, the projectile that had penetrated his sclera was likely an inert object or an encapsulated metal as a result of giant cell reaction. A retained encapsulated metallic IOFB can be asymptomatic, as the encapsulation limits the free passage of active metal ions [2]. Moreover, metallic IOFBs are asymptomatic if made of an inert metal, such as silver, aluminum, platinum, or gold [4]. A study of 10 eyes harboring metallic IOFBs for nine to 46 years found that amplitude on electroretinography was reduced in only one eye, with this reduction clearly related to the toxic effect of the IOFB [6]. Iatrogenic retinal detachment, which can be induced during vitrectomy and surgical manipulation of IOFBs, can predict visual outcomes. A prospective study of four eyes that underwent removal of inert IOFBs found that one had a poor visual outcome due to concomitant endophthalmitis and retinal detachment [7]. Our patient should avoid magnetic resonance imaging as the IOFB could be a ferromagnetic metal that can undergo displacement when exposed to a magnetic field, causing damage to the adjacent intraocular structures [8].

## Conclusions

The decision to remove a missed retained IOFB is complex and depends on multiple factors, including surgical difficulty and the composition, size, and location of the retained foreign body. Removal should be weighed against the possible serious complications of intraocular surgery. If removal is surgically difficult, or the retained material is inert, patients can be managed conservatively with regular monitoring.
